# Site-specific analysis of gene expression in early osteoarthritis using the Pond-Nuki model in dogs

**DOI:** 10.1186/1749-799X-1-8

**Published:** 2006-10-10

**Authors:** Aaron M Stoker, James L Cook, Keiichi Kuroki, Derek B Fox

**Affiliations:** 1 The Comparative Orthopaedic Laboratory, University of Missouri Columbia, 379 E Campus Dr, Columbia, MO, USA; 2 Kansas State University Veterinary Diagnostic Laboratory, Kansas State University, 1800 Denison Avenue, Manhattan, KS, USA

## Abstract

**Background:**

Osteoarthritis (OA) is a progressive and debilitating disease that often develops from a focal lesion and may take years to clinically manifest to a complete loss of joint structure and function. Currently, there is not a cure for OA, but early diagnosis and initiation of treatment may dramatically improve the prognosis and quality of life for affected individuals. This study was designed to determine the feasibility of analyzing changes in gene expression of articular cartilage using the Pond-Nuki model two weeks after ACL-transection in dogs, and to characterize the changes observed at this time point.

**Methods:**

The ACL of four dogs was completely transected arthroscopically, and the contralateral limb was used as the non-operated control. After two weeks the dogs were euthanatized and tissues harvested from the tibial plateau and femoral condyles of both limbs. Two dogs were used for histologic analysis and Mankin scoring. From the other two dogs the surface of the femoral condyle and tibial plateau were divided into four regions each, and tissues were harvested from each region for biochemical (GAG and HP) and gene expression analysis. Significant changes in gene expression were determined using REST-XL, and Mann-Whitney rank sum test was used to analyze biochemical data. Significance was set at (p < 0.05).

**Results:**

Significant differences were not observed between ACL-X and control limbs for Mankin scores or GAG and HP tissue content. Further, damage to the tissue was not observed grossly by India ink staining. However, significant changes in gene expression were observed between ACL-X and control tissues from each region analyzed, and indicate that a unique regional gene expression profile for impending ACL-X induced joint pathology may be identified in future studies.

**Conclusion:**

The data obtained from this study lend credence to the research approach and model for the characterization of OA, and the identification and validation of future diagnostic modalities. Further, the changes observed in this study may reflect the earliest changes in AC reported during the development of OA, and may signify pathologic changes within a stage of disease that is potentially reversible.

## Background

Osteoarthritis (OA) is a progressive and debilitating disease that may take years to clinically manifest in affected individuals [1,2]. OA often progresses from a focal loss of articular cartilage integrity to a complete loss of joint structure and function. Currently, there is not a cure for OA, and available treatments only slow the progression of disease. Early diagnosis with initiation of treatment may dramatically improve the prognosis and quality of life for affected individuals [3-5]. Radiographic evaluation and advanced imaging modalities such as computed tomography and standard magnetic resonance imaging can be helpful in determining extent and severity of the disease process[6-9]. However, no imaging techniques currently provide definitive data for early diagnosis, accurate monitoring of response or progression, or prognostication in OA. Other techniques for early, more sensitive diagnoses are being developed, including serum and synovial fluid biomarkers, biomechanical testing of articular cartilage tissue, and optical coherence tomography[10-12]. However, none provides data for definitive diagnosis of OA prior to irreversible pathology. Further, the earliest stages of OA are poorly characterized and methods for determining a definitive diagnosis of OA in potentially reversible stages of disease are not currently available to the authors' knowledge.

It is clear that during the development of OA, cartilage tissue metabolism shifts from extracellular matrix (ECM) homeostasis to degradation. Further, once articular cartilage (AC) is irreversibly damaged, as in OA, regenerative healing does not occur and function is impaired[13,14]. The ECM of normal articular cartilage can remodel in response to applied load, and matrix molecules are degraded and replaced during the process of physiologic ECM turnover. Therefore, it appears that AC does have some capacity to repair damage to the ECM. What is not known is at what point the degree of damage to the ECM is beyond the capabilities of tissue repair mechanisms. Further, and perhaps more importantly, methods for diagnosing the point at which recovery is no longer possible are not known.

Two potential factors that may influence the "point of no return" in the progression of OA are chondrocyte viability and phenotype. During the development of OA, there is often a focal increase in cell death[15-18]. Since it is theorized that each chondrocyte is responsible for the maintenance of the ECM surrounding it, and that matrix molecules produced in one region of the tissue have a very limited ability to traverse the tissue, the focal loss of viable cells could be partially responsible for the tissues inability to repair minor damage [19]. In addition, surviving chondrocytes undergo a phenotypic shift that includes expression of inappropriate matrix molecules[20-22], decreased sensitivity to insulin like growth factor-1 (IGF-1)[23], increased expression of vascular endothelial growth factor (VEGF) and VEGF receptor[24,25], decreased expression of chondromodulin-I (ChM-I)[24], altered interleukin (IL)-4 signaling[25], and altered integrin-dependent mechanotransduction pathways[26]. However, the exact timing and complete nature of phenotypic changes in osteoarthritic chondrocytes and the associated alterations in gene expression are not known at this time.

In order to understand the earliest stages in the pathogenesis of OA, studies need to be designed that examine changes that occur in AC prior to irreversible damage. Animal models have been developed which allow longitudinal study of OA with a known time of initiation[27-33]. For the present study, the Pond-Nuki model of OA[34] was chosen. Two weeks after surgery the animals were euthanatized and AC from defined regions of the femoral condyles and tibial plateaus of both the operated and non-operated control stifles was analyzed for histologic, biochemical, and molecular measures of cell and matrix changes.

This study was designed to determine the feasibility of analyzing changes in gene expression of articular cartilage two weeks after ACL-transection. The specific aims of this study were to determine if changes in relevant gene expression could be observed two weeks after ACL transection in dogs which correlate to future pathology as indicated by historical data in this model; determine if articular cartilage from different regions of the joint surface have unique changes in relative gene expression levels in response to ACL transection; and characterize the changes in gene expression at this time point. It was hypothesized that significant increases in gene expression for degradative enzymes (MMPs and ADAMTS) as well as inflammatory indicators (INOS and COX-2) would be observed in those regions of the articular cartilage which historically undergo gross and histologic changes after ACL-X, while the expression for antidegradative (TIMPs) and matrix molecules (Col 1, Col 2, Aggrecan) would be unchanged or decreased in these same regions. A potential pattern of regional differential gene expression was observed in this study indicative of increased inflammatory, degradative, and repair/remodeling response with the articular cartilage tissue. These data will be used in future studies aimed at better characterizing the changes that occur in the joint during the development of OA and for studies aimed at developing and evaluating diagnostic, preventative, and therapeutic strategies for OA.

## Methods

### Pond-Nuki model

All procedures were approved by the University of Missouri Animal Care and Use Committee. Adult (2–4 years of age), hound-mix (mean weight = 27.6 Kg, range: 24.3–33.1 kg)) research dogs (n = 4) were premedicated, anesthetized, and prepared for aseptic surgery of one randomly assigned stifle. Routine craniolateral and craniomedial arthroscope and instrument portals were established and the anterior cruciate ligament was completely transected arthroscopically. Complete transection of the ACL was confirmed by visual observation and palpation of anterior tibial translocation. Analgesics (morphine or aspirin) were administered to the dogs at the time of extubation, and then as necessary to control signs of pain (aspirin was discontinued 48 hours post-op). The dogs were recovered and returned to their individual kennels. The dogs were allowed to use the affected limb in a 10 × 10 foot kennel. In addition, the dogs were walked on a leash twice daily for 10 minutes at a pace that ensured use of all four limbs.

Two weeks after surgery, the dogs were euthanatized by intravenous overdose of a barbiturate. After euthanasia, both stifles of each dog were carefully disarticulated and examined. The menisci were examined and any gross meniscal pathology was recorded. The tibial plateau and femoral condyles were photographed. All articular surfaces were painted with India ink, washed after 60 seconds with tap water, and photographed. If staining was observed, then unexposed radiographic film was placed over each condyle and plateau, and cut to match the surface area of the condyle. The areas of India ink staining were outlined using a permanent marker. Tracings of the India ink-stained tibial and femoral condyles were evaluated without knowledge of dog number or treatment group. The tracings were scanned using a computer software program and percentage of the total area of the tibial and the femoral condyles that stained calculated and recorded as % area of cartilage damage (%ACD). The %ACD was determined for the tibial and femoral condyles, separately and together, for each dog. Tissue was harvested from the affected and unoperated contralateral limb as described below.

### Tissue harvest

**Figure 1:**
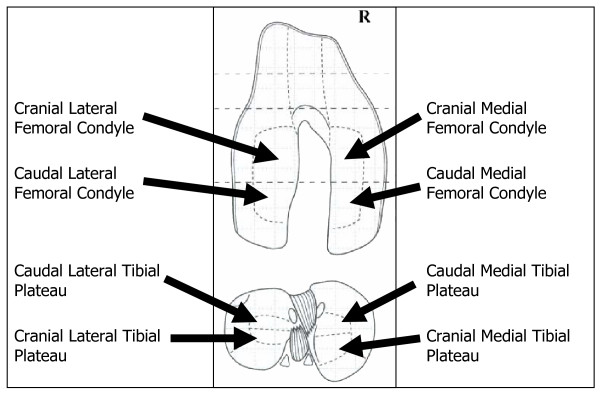
**Regions of the Femoral Condyle and Tibial Plateau utilized for tissue harvest**. Tissue samples were taken from each region for biochemical and gene expression analysis.

Full-thickness articular cartilage samples were collected from the cranial medial femoral condyle (CrMFC), caudal medial femoral condyle (CaMFC), cranial lateral femoral condyle (CrLFC), caudal lateral femoral condyle (CaLFC), cranial medial tibial plateau (CrMTP), caudal medial tibial plateau (CaMTP), cranial lateral tibial plateau (CrLTP), and caudal lateral tibial plateau (CaLTP) from affected and contralateral control limb of two dogs (Figure 1). One sample per region per animal was evaluated for relative gene expression level and matrix biochemical composition. Cartilage samples collected for gene expression analysis were snap-frozen in liquid nitrogen and stored at -80°C. Cartilage samples collected for biochemical assays were weighed to determine wet weight and stored at -20°C. The affected and contralateral tibial plateau and femoral condyles from the other two dogs were harvested, and serial sagittal sections were made from medial to lateral to include articular cartilage and subchondral bone. The sections were fixed in 10% buffered formalin and decalcified by emersion in Surgipath Decalsifier II at room temperature for 24 hours. The fixed tissues were paraffin embedded, and 5-micron sections were cut and stained with hematoxylin and eosin (H&E) and toluidine blue for subjective histologic assessment.

### Papain digestion of tissues

Articular cartilage samples were digested overnight at 65°C using 500 μl of papain digestion buffer (20 mM sodium phosphate buffer, 1 mM EDTA, 300 μg/ml (14 U/mg) of papain, and 2 mM DTT), and then stored at -20°C until analyzed further.

### Glycosaminoglycan (GAG) assay

Total sulfated GAG content was determined using the dimethylmethylene blue (DMMB) assay[35]. The GAG content of each sample was determined by adding 245 μl of DMMB to 5 μl of each papain digested sample, and absorbance was determined at 530 nm. Known concentrations of chondroitin sulfate (2.5 μg to .3125 μg)(Sigma, St. Louis, MO) were used to create a standard curve. Results were standardized to the wet weight of each tissue and reported as μg/mg tissue wet weight.

### Hydroxyproline (HP) assay

Total collagen content was determined using a colorimetric assay to measure the HP content[36]. The assay was modified to a 96-well format. A 50 μl sample from the papain digested tissues was mixed with an equal volume of 4N sodium hydroxide in a 1.2 ml deep-well 96-well polypropylene plate. The plate was covered with silicon sealing mat, a polypropylene cover was placed on top of the mat, and the plates were stacked. The plates were sealed by compression with a C-Clamp, and autoclaved at 120°C for 20 min to hydrolyze the sample. Chloramine T reagent (450 μl) was mixed with each sample, and incubated for 25 min at 25°C. Ehrlich aldehyde reagent (450 μl) was mixed with each sample and incubated for 20 min at 65°C to develop the chromophore. Known concentrations of HP (Sigma, St. Louis, MO) were used to construct a standard curve (20 μg to 2 μg). A portion (100 μl) of each sample was transferred to a new 0.2 ml 96-well plate, and absorbance read at 550 nm. Values obtained were standardized to the wet weight of the cartilage explant and reported as mg/mg tissue wet weight.

### RNA extraction

Total RNA was extracted using the Trispin method[37]. Explants were disrupted in liquid nitrogen utilizing a tissue crusher, homogenized in 1 ml of TRIzol (Invitrogen, Carlsbad, CA) using a mini-beadbeater (Biospec Products, Bartlesville, OK) and 2 mm zirconia beads (Biospec Products, Bartlesville, OK). The homogenate was transferred to a new tube, and insoluble material was pelleted by centrifugation. The supernatant was transferred to a new tube and Chloroform (200 μl) was added to each sample. The organic and aqueous phases were separated by high speed centrifugation. The upper aqueous phase was transferred to a new tube and ethanol (ETOH) was added to the aqueous phase to a final concentration of 35%, mixed by vortexing, and passed through an RNeasy mini-column (Qiagen Valencia, CA) to bind the RNA. The column was washed with wash buffer; contaminating DNA was digested on the column with DNase 1 (Qiagen Valencia, CA); and the column was washed three more times. RNA was eluted off the column using 30 μl of RNase free water. The yield of extracted total RNA was determined by measuring absorbance at 260 nm, and purity was assessed using the ratio of absorbance readings at 260 nm to 280 nm. The average RNA yield was approximately 1 μg of total RNA per sample.

### Real Time RT-PCR

#### Reverse transcription

Reverse transcription was performed using Stratascript™ reverse transcriptase (Stratagene, La Jolla, CA), according to the manufacturer's protocol. Total RNA (500 ng) from each sample was mixed with 10 pM of random hexamers and RNase-free water to a final volume of 16 μl. The mixture was then incubated at 68°C for 5 minutes and transferred to ice for 3 minutes. After incubation on ice 4 μl of the reaction mixture, containing 2 μl of the 10X Stratascript™ buffer, 1 μl of 10 mM dNTPs, and 1 μl of the Stratascript™ enzyme, was added to each sample. The samples were then incubated in a PE GeneAmp 9700 for 90 min at 45°C and then held at 4°C. The RT reaction was diluted to 200 μl with RNase free water and stored at -20°C until analyzed by real-time PCR.

#### Real-Time PCR

Real-Time PCR was performed using the QuantiTect™ SYBR^® ^Green PCR kit (Qiagen Valencia, CA) and the Rotor-Gene 3000™ real-time PCR thermalcycler (Corbett Research, Sydney, Australia). The reaction mixture consisted of 4 μl of diluted cDNA, 0.3 μM of forward and reverse primers (1 μl each), 10 μl of the 2X QuantiTect™ SYBR^® ^green master mix, 0.1 μl of HK-UNG (Epicentre, Madison, WI), and 4 μl of RNase-free water for each sample for a total volume of 20 μl. The PCR profile consisted of 5 min at 35°C; 15 min at 94°C; 50 cycles of 5 seconds (sec) at 94°C (melting), 10 sec at 57°C (annealing), and 15 sec at 72°C (extension); and a melt curve analysis from 69 to 95°C. Fluorescence was detected during the extension step of each cycle and during the melt curve analysis at 470 nm/510 nm (excitation/emission) for SYBR^® ^green. Melt curve analysis was performed to ensure specific amplification. Take off point (C_t_) and amplification efficiency were determined using the comparative quantification analysis provided with the Rotor-Gene software. Melt curve analysis were performed using the melt curve analysis function provided with the Rotor-Gene software. Canine specific primers (Table 1) were developed for glyceraldehyde-3-phosphate dehydrogenase (GAPDH), collagen (COL) 1, COL 2, aggrecan, tissue inhibitor of matrix metalloproteinases (TIMP)-1, TIMP-2, matrix metalloproteinase (MMP)-1, MMP-3, MMP-13, Aggrecanase-1 (ADAMTS4), Aggrecanase-2 (ADAMTS5), inducible nitric oxide synthase (INOS), and cyclooxygenase-2 (COX-2) using canine sequences available in Genbank. If canine specific sequence was not available, then degenerate primers were developed using sequence data available for multiple species. The degenerate primers were then used to amplify the canine sequence by standard PCR. The amplified section was sequenced, compared to the Genbank database by BLAST to determine specificity, and canine specific Real-Time PCR primers were developed from the obtained sequence.

**Table 1:** Primer sets used for Real-Time PCR analysis

Gene	Orientation	Primer Sequence	Amplicon Size	Melt Temp
GAPDH	FOR	GTGACTTCAACAGTGACACC	152	84.7
	RC	CCTTGGAGGCCATGTAGACC		
Aggrecan	FOR	ATCGAAGGGGACTTCCGCTG	106	84.5
	RC	ATCACCACACAGTCCTCTCCG		
COL 2	FOR	GGCCTGTCTGCTTCTTGTAA	197	83.3
	RC	ATCAGGTCAGGTCAGCCATT		
COL 1	FOR	TGCACGAGTCACACTGGAGC	124	85.5
	RC	ATGCCGAATTCCTGGTCTGG		
TIMP 1	FOR	GCAGAAGTCAACCAGACCGA	311	86.2
	RC	GCAAGTATCCGCAGACGCTC		
TIMP 2	FOR	AACGGCAAGATGCACATCAC	142	85.5
	RC	ATATAGCACGGGATCATGGG		
INOS	FOR	GCTATGCTGGCTACCAGATG	139	88.3
	RC	ATCAGCCTGCAGCACCAGAG		
COX-2	FOR	ACACTCTACCACTGGCATCC	196	83.5
	RC	GCTACTTGTTGTACTGCAGC		
MMP-1	FOR	CCTAGAACCGTGAAGAGCAT	150	80
	RC	CAGGAAAGTCAGCTGCTATC		
MMP 3	FOR	ATGGCATCCAGTCCCTGTAT	161	86.5
	RC	AAAGAACAGGAACTCTCCCC		
MMP 13	FOR	TCTGGTCTTCTGGCTCATGC	141	82.7
	RC	GGTCAAGACCTAAGGAGTGG		
ADAMTS4	FOR	CATCACTGAGTTCCTGGACA	106	84.5
	RC	CGATCAGCGTCATAGTCCTT		
ADAMTS5	FOR	TGACTTCTTGCATGGCATGG	120	81.5
	RC	CTGGCATGGCTGGTGACTGA		

Canine primer sets used for real-time PCR analysis. The annealing temperature used for all analysis was 57°C. The melt temperature for the amplicon was obtained from the Rotorgene software and is indicative of a specific PCR reaction.

### Histologic analysis

Histologic sections from all sites of both ACL-X and control stifles were stained with hematoxylin and eosin (H&E) and toluidine blue. Sections were evaluated subjectively by one investigator blinded to sample group or number. Subjective assessment included histologic evidence of cell viability, cell density, and cell morphology; articular cartilage surface architecture; and proteoglycan staining characteristics.

### Statistical analysis

Relative levels of gene expression were determined using Q-Gene[38] and the housekeeping gene GAPDH as an internal standard. To assess for differences in gene expression, the non-parametric relative expression statistical tool (REST-XL)[39,40] was used. Differences in gene expression were considered significant when p < 0.05 and the difference in expression between ACL-X and contralateral limbs was >2X for both animals. The statistical software SigmaStat 2.03 (Jandel Scientific, San Rafael, CA) was used to compare data from biochemical assays. Data from each sample group were combined and a Mann-Whitney Rank Sum test was performed to determine significant differences between ACL-X and Control tissues for biochemical analyses. Significance was set a p < 0.05.

## Results

### Gross and histologic analysis

No AC damage was present on the femoral condyles or tibial plateaus in any of the ACL-X or control stifles based on India ink staining (data not shown). No histologic evidence consistent with degenerative or osteoarthritic change was noted in any section based on subjective evaluation (data not shown).

### Biochemical analysis

**Figure 2:**
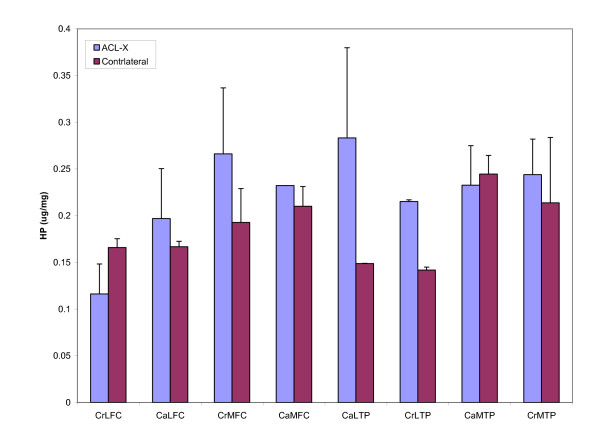
**Hydroxyproline content of cartilage by region**. The HP content of each cartilage region from the ACL-X joint was compared to the corresponding region in the contralateral control joint. Significant differences were not observed in the HP content of the tissues between ACL-X and control joints for any of the regions tested. Error bars indicate standard error of the mean. Values are μg of HP/mg of tissue wet weight.

**Figure 3:**
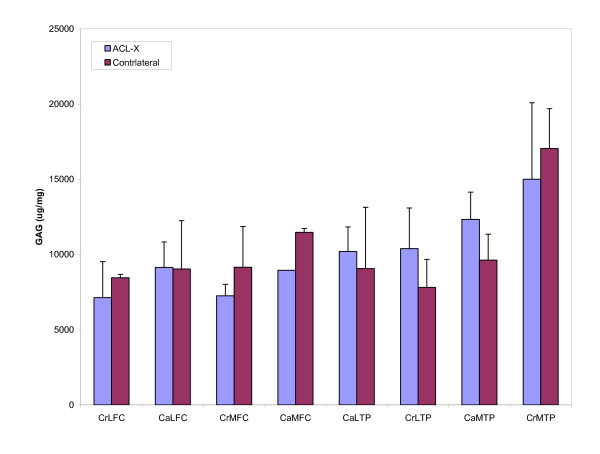
**Sulfated glycosaminoglycan content of cartilage by region**. The GAG content of each cartilage region from the ACL-X joint was compared to the corresponding region in the contralateral control joint. Significant differences were not observed in the GAG content of the tissues between ACL-X and control joints for any of the regions tested. Error bars indicate standard error of the mean. Values are μg of GAG/mg of tissue wet weight.

No significant differences in levels of total glycosaminoglycans (p = 0.21, power = 0.16) or hydroxyproline (p = 0.21, power = 0.16) were observed between ACL-X and control stifles in any region studied (Figures 2 and 3). However the powers of the analyses were lower than 0.8, and therefore should be interpreted with caution.

### Gene expression analysis

**Figure 4:**
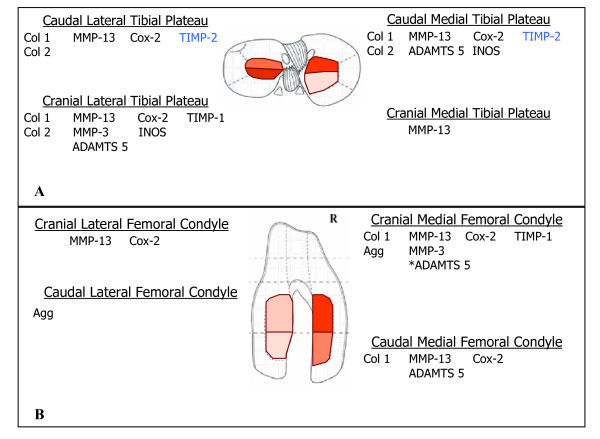
**Differentially expressed genes by region**. Graphical representation of genes differentially expressed by region in the tibial plateau (A) and femoral condyle (B). Increasing shade of red indicates an increased number of genes differentially expressed in that region. * ADAMTS 5 gene expression approached significance (*p *= .055)

Significant differences (p < 0.05) in gene expression between ACL-X and control stifles were observed in every region analyzed (Table 2 and 3, Figure 4), and each region exhibited a unique gene expression pattern. The CrMFC, CaMFC, CaMTP, CaLTP, and CrLTP regions exhibited the greatest number of differentially expressed genes when comparing ACL-X to control tissues. The CrMTP exhibited the least number of genes exhibiting differential expression, followed by the CrLFC and CaLFC.

**Table 2:** Differentially expressed genes in the ACL-X knee by region in the femoral chondyle

**p value**	**ACL-X**	**Control**	**CrMFC**	**CrLFC**	**ACL-X**	**Control**	**p value**
<0.05	11.1 ± 1.71	0.57 ± 0.27	COL 1	MMP 13	0.096 ± 0.061	0.006 ± 0.002	<0.05
<0.05	859 ± 229	298 ± 212	Aggrecan	COX-2	0.017 ± 0.012	0.001 ± 0.001	<0.05
<0.05	0.075 ± 0.032	0 ± 0	MMP 13				
<0.05	0.106 ± 0.092	0.001 ± 0.001	COX-2				
<0.05	50.8 ± 18.8	16.8 ± 11.9	TIMP-1				
<0.05	10.4 ± 6.8	0.744 ± 0.224	MMP 3				
0.0555	0.853 ± 0.714	0.01 ± 0.006	ADAMTS 5				

**p value**	**ACL-X**	**Control**	**CaMFC**	**CaLFC**	**ACL-X**	**Control**	**p value**

<0.05	709 ± 659	4.32 ± 0.35	COL 1	Aggrecan	370 ± 262	98 ± 23	<0.05
<0.05	1.224 ± 1.189	0.006 ± 0.001	MMP 13				
<0.05	0.009 ± 0.005	0.001 ± 0.001	COX-2				
<0.05	0.055 ± 0.028	0.016 ± 0.013	ADAMTS 5				

Differentially expressed genes in the ACL-X knee by region in the femoral chondyle compared to the contralateral normal control. All genes listed were up regulated in the ACL-transected side. Values listed are the mean relative level of expression for each gene (± standard error) compared to the house keeping gene GAPDH. The increase in ADAMTS 5 gene expression in the CrMFC approached significance and was included in the table. Significant differences were determined using REST-XL, and relative expression levels were determined using Q-Gene.

**Table 3:** Differentially expressed genes in the ACL-X knee by region in the tibial plateau

**p value**	**ACL-X**	**Control**	**CrMTP**	**CrLTP**	**ACL-X**	**Control**	**p value**
<0.05	0.093 ± 0.02	0.018 ± 0.018	MMP 13	COL 1	5.46 ± 3.96	0.48 ± 0.08	<0.05
				COL 2	1022 ± 651	254 ± 84	<0.05
				MMP 13	0.147 ± 0.012	0 ± 0	<0.05
				INOS	0.362 ± 0.065	0.04 ± 0.04	<0.05
				COX-2	0.007 ± 0.005	0 ± 0	<0.05
				TIMP-1	35.3 ± 4.6	8.4 ± 5.4	<0.05
				MMP 3	2.428 ± 1.272	0.587 ± 0.18	<0.05
				ADAMTS 5	0.132 ± 0.05	0.024 ± 0.024	<0.05

**p value**	**ACL-X**	**Control**	**CaMTP**	**CaLTP**	**ACL-X**	**Control**	**p value**

<0.05	378.6 ± 321.42	9.05 ± 7.08	COL 1	COL 1	78.85 ± 43.79	1.98 ± 1.72	<0.05
<0.05	2787 ± 1919	594 ± 151	COL 2	COL 2	1926 ± 939	708 ± 49	<0.05
<0.05	0.074 ± 0.059	0.007 ± 0.007	MMP 13	MMP 13	0.05 ± 0.002	0.011 ± 0.011	<0.05
<0.05	0.051 ± 0.044	0.003 ± 0.001	COX-2	COX-2	0.019 ± 0.006	0.002 ± 0.002	<0.05
<0.05	0.189 ± 0.019	0.088 ± 0.002	INOS				
<0.05	0.023 ± 0.011	0.002 ± 0.002	ADAMTS 5				
<0.05	1.4 ± 0.07	3.71 ± 0.08	TIMP-2	TIMP-2	0.99 ± 0.02	2.7 ± 0.8	<0.05

Differentially expressed genes in the ACL-transected knee by region in the tibial plateau compared to the contralateral normal control. All genes listed were up regulated in the ACL-transected stifle except TIMP-2, which was down regulated in the ACL-transected stifle. Values listed are the mean relative level of expression (± standard error) for each gene compared to the house keeping gene GAPDH. Significant differences were determined using REST-XL, and relative expression levels were determined using Q-Gene.

The only gene analyzed found to have a significant (p < 0.05) decrease in expression in ACL-X AC was TIMP-2 and this was only noted in the CaLTP and CaMTP regions. MMP-13 gene expression was significantly (p < 0.05) higher for ACL-X cartilage in all regions except the CaLFC, and had the highest fold increase in relative gene expression. Regional increases in TIMP-1, COX-2 and INOS were detected in ACL-X cartilage, as well as the degradative enzymes ADAMTS5 and MMP-3. Aggrecan expression was increased in the CaLFC and the CrMFC, while Collagen 2 expression was increased in the CaLTP, CaMTP, and CrLTP of ACL-X stifles. Col 1 gene expression was upregulated in regions of both the femoral condyles and the tibial plateaus in ACL-X stifles. Gene expression for MMP-1 and ADAMTS 4 were highly variable and not significantly different between ACL-X and control tissues (data not shown).

## Discussion

The results presented in this work indicate that relevant changes in chondrocyte gene expression can be detected in dogs two weeks after complete transection of the ACL. To the authors' knowledge, this is the earliest time point reported for site-specific gene expression analysis in dogs using this model. Previous chondrocyte gene expression analysis using the ACL-X transection model within a similar time frame was performed in rabbits[41,42]. The Pond-Nuki model in dogs appears to provide a more appropriate model for OA in humans with respect to tissue involvement, nature of pathology, and diagnostic findings, as well as an extensive historical data base for comparison[34,43-46]. Therefore, we chose to use this model in dogs for molecular analysis of specific regions of articular cartilage during the early stages of OA, prior to gross or histologic evidence of pathology in an attempt to produce the most comprehensive, translational, and clinically relevant data possible.

In the present study, AC from both the tibial plateau (TP) and femoral condyles (FC) showed no evidence of osteoarthritis based on gross, histologic, and biochemical assessments. The lamina splendens was not disrupted in any location based on lack of India ink staining, indicating that AC surface integrity was maintained for two weeks in the dogs in this study. Histologically, all AC subjectively appeared to have normal cell morphology, density, and distribution and ECM architecture and composition. Further, there were not significant differences in proteoglycan or collagen levels between ACL-X and control stifles, as determined by total GAG and HP content. When considered together, these data indicate that AC in the ACL-X stifles was still "normal" by phenotypic measures 2 weeks after ACL-X transection. The lack of gross, histologic, or biochemical changes in AC supports previous work that indicates that observable changes in AC do not occur prior to 4 weeks after ACL-X in dogs[47,48].

The regional changes in gene expression observed in this study suggest that focal biochemical, histological, and gross changes in specific areas of AC consistently seen in OA begin with alterations in gene expression. The medial FC had a higher number of genes with significant changes in relative expression levels compared to the lateral FC after ACL-X. These data indicate that in this model the medial FC is more affected by the insults to the joint induced by transection of the ACL, which is in agreement with previous studies in dogs[49] and other species[42]. Differential gene expression data from the TP indicate that tibial cartilage is more diffusely affected than femoral articular cartilage after ACL transection. The CrLTP, the CaLTP, and the CaMTP were most affected by transection of the ACL with respect to changes in gene expression in this study. These data are also in agreement with previous studies using the Pond-Nuki model which reported lesion formation in both lateral and medial aspects of tibial cartilage[42,49]. Continued research is required to determine if the regional differential gene expression profile observed in this study occur consistently, and if the potential regional gene expression profile observed at this time point can accurately predict phenotypic changes that consistently occur at later time points during the progression of OA. Further, two weeks after arthroscopic ACL-X surgery inflammatory processes associated with healing would be expected. The increased expression of COX-2 seen in many regions of AC may indicate that inflammation from surgery is affecting the tissues, and therefore likely affecting the gene expression changes observed in this study. The roles of surgery induced inflammation and post operative healing on regional changes in chondrocyte gene expression, must be further investigated. On going studies in our laboratory include sham operated dogs as well as posterior cruciate ligament transected dogs to distinguish the affects of these variables on the nature, severity, and progression of joint pathology.

The overall pattern of gene expression observed in the ACL-X AC indicated a potential shift in cellular metabolism consistent with early osteoarthritis. Increases in COX-2 and INOS in regions most affected by joint instability were consistent with signaling events that typically occur in OA joints[50,51]. The concurrent and often co-localized increases in gene expression of COL 2, aggrecan, MMP 13, and ADAMTS 5 seen in this study closely match the elevated synthetic and degradative changes reported to occur at the protein level in early OA[41,52-54].

Interestingly, three genes, MMP 13, COX-2, and COL 1, were upregulated in all regions of ACL-X cartilage that had relatively high numbers of differentially expressed genes. Further, the present study provides data regarding the potential hierarchy of expression of these three genes. The CrLFC showed upregulation of both MMP 13 and COX-2, while the CaMTP showed upregulation of MMP 13. This could indicate that during the early development of OA, MMP 13 gene expression is affected first, followed by COX-2, and then COL 1. Based on this consistent upregulation of these 3 important genes in cartilage metabolism, it seems plausible that together these genes may be useful markers for diagnosis and monitoring of disease progression in OA. If this possibility can be validated, assessment of these markers could prove to be a valuable tool as a diagnostic test for early OA.

## Conclusion

Though the number of animals analyzed in this study was considered by the authors to be too small (n = 2 for all assessments) to make definitive conclusions with respect to pathophysisology of early OA or clinical relevance of these data, the findings from this study lend credence to the research approach and use of this model for the characterization of OA, and the identification and validation of future diagnostic modalities. Further, the changes observed in this study may reflect the earliest changes in AC reported during the development of OA, and may reflect pathologic changes within a stage of disease that is potentially reversible. By investigating specific regions that have the highest number of differentially expressed genes, it is possible that potential diagnostic markers will be identified that can be utilized to diagnose OA early enough to prevent the progression of the disease, or at least optimally minimize the clinical signs and symptoms of OA. Further, potential targets for treatment could be identified. Ongoing research in our laboratory using this experimental approach is focused on identifying and developing diagnostic methods and markers, as well as strategies for prevention and treatment of OA in the earliest stages of disease.

## Competing interests

The author(s) declare that they have no competing interests.

## Authors' contributions

AS: study design, sample harvesting, sample processing, acquisition of data, analysis and interpretation of data, writing of manuscript. KK: animal care, sample harvesting, acquisition of data, editing of manuscript. DF: animal care, surgical procedures, sample harvesting, editing of manuscript. JC: animal care, surgical procedures, sample harvesting, analysis and interpretation of data, writing of manuscript.
